# In vitro evaluation of microencapsulated organic acids and pure botanicals as a supplement in lactating dairy cows diet on in vitro ruminal fermentation

**DOI:** 10.1093/tas/txad099

**Published:** 2023-08-21

**Authors:** Richard R Lobo, Michael Watson, James R Vinyard, Mikayla L Johnson, Aneesa Bahmam, Szu-Wei Ma, Gamonmas Dagaew, Phussorn Sumadong, Efstathios Sarmikasoglou, Ester Grilli, Jose A Arce-Cordero, Antonio P Faciola

**Affiliations:** Department of Animal Sciences, University of Florida, Gainesville, FL 32608, USA; Department of Animal Sciences, University of Florida, Gainesville, FL 32608, USA; Department of Animal Sciences, University of Florida, Gainesville, FL 32608, USA; Department of Animal Sciences, University of Florida, Gainesville, FL 32608, USA; Department of Animal Sciences, University of Florida, Gainesville, FL 32608, USA; Department of Animal Sciences, University of Florida, Gainesville, FL 32608, USA; Department of Animal Science, Khon Kaen University, Khon Kaen 40002, Thailand; Department of Animal Science, Khon Kaen University, Khon Kaen 40002, Thailand; Department of Animal Sciences, University of Florida, Gainesville, FL 32608, USA; Dipartimento di Scienze Mediche Veterinarie, Università di Bologna, Bologna 40064, Italy; Vetagro S.p.A., Reggio Emilia 42124, Italy; Escuela de Zootecnia, Universidad de Costa Rica, San José 11501-2060, Costa Rica; Department of Animal Sciences, University of Florida, Gainesville, FL 32608, USA

**Keywords:** citric acid, sorbic acid, thymol, vanillin

## Abstract

The utilization of microencapsulated organic acids and pure botanicals (**mOAPB**) is widely used in the monogastric livestock industry as an alternative to antibiotics; in addition, it can have gut immunomodulatory functions. More recently, an interest in applying those compounds in the ruminant industry has increased; thus, we evaluated the effects of mOAPB on ruminal fermentation kinetics and metabolite production in an in vitro dual-flow continuous-culture system. For this study, two ruminal cannulated lactating dairy Holstein cows were used as ruminal content donors, and the inoculum was incubated in eight fermenters arranged in a 4 × 4 Latin square design. The basal diet was formulated to meet the nutritional requirements of a 680-kg Holstein dairy cow producing 45 kg/d of milk and supplemented with increasing levels of mOAPB (0; 0.12; 0.24; or 0.36% of dry matter [**DM**]), which contained 55.6% hydrogenated and refined palm oil, 25% citric acid, 16.7% sorbic acid, 1.7% thymol, and 1% vanillin. Diet had 16.1 CP, 30.9 neutral detergent fiber (**NDF**), and 32.0 starch, % of DM basis, and fermenters were fed 106 g/d split into two feedings. After a 7 d adaptation, samples were collected for 3 d in each period. Samples of the ruminal content from the fermenters were collected at 0, 1, 2, 4, 6, and 8 h postmorning feeding for evaluation of the ruminal fermentation kinetics. For the evaluation of the daily production of total metabolites and for the evaluation of nutrient degradability, samples from the effluent containers were collected daily at days 8 to 10. The statistical analysis was conducted using MIXED procedure of SAS and treatment, time, and its interactions were considered as fixed effects and day, Latin square, and fermenter as random effects. To depict the treatment effects, orthogonal contrasts were used (linear and quadratic). The supplementation of mOAPB had no major effects on the ruminal fermentation, metabolite production, and degradability of nutrients. The lack of statistical differences between control and supplemented fermenters indicates effective ruminal protection and minor ruminal effects of the active compounds. This could be attributed to the range of daily variation of pH, which ranged from 5.98 to 6.45. The pH can play a major role in the solubilization of lipid coat. It can be concluded that mOAPB did not affect the ruminal fermentation, metabolite production, and degradability of dietary nutrients using an in vitro rumen simulator.

## Introduction

Antimicrobials’ discovery and introduction in human health science were first described in the 1940s and shortly after introduced to veterinary practices (Witte, 2000; Kirchhelle, 2018). Early reports on the utilization of antimicrobials to reduce intestinal infection in chickens and pigs with sulfonamide and streptomycin being used as feed supplements generated beneficial effects, such as reducing mortality and improving body weight (Moore and Everson, 1946; Luecke, 1950), and expanded quickly to other livestock species like cattle. It is estimated that the annual worldwide cattle consumption of antibiotics is around 45 mg/kg of body weight and that in 2030 such consumption would increase by 67% compared to 2010 (Van Boeckel et al., 2015).

One of the main concerns of antibiotic utilization as a gut microbiome modulator in livestock animals is the development of resistance of microorganisms to antimicrobial agents (Landers et al., 2012; Murray et al., 2022). Therefore, the search for alternatives to antibiotics has increased in the past decades, and one of the most promising alternatives is the use of organic acids and pure botanicals. These are biochemical compounds from the plant and microbial metabolism that can be extracted and used as supplements in the diet of livestock animals (Sang and Blecha, 2015).

Blends of organic acids (**OA**) and pure botanicals (**PB**) have been used in swine and poultry nutrition successfully; however, their use in ruminants has been limited because of the need to escape ruminal degradation in order to make them available at intestinal level. Microencapsulation of OA and PB would potentially minimize or eliminate ruminal effects and ruminal degradation, allowing these compounds to be available for digestion and absorption in the small intestine. Microencapsulated blends of organic acids and plant botanicals (**mOAPB**) have been used in the livestock feed industry as an alternative to growth-promoting antibiotics (EFSA, 2012; Tugnoli et al., 2020), due to their effectiveness as antimicrobials and gut immunomodulators (Grilli et al., 2013; Hutchens et al., 2021). More recently, studies reported that mOAPB are able to partially restore feed intake of lactating cows and calves during heat stress (Fontoura et al., 2022, 2023); however, it is not well characterized the mode of action of mOAPB that stimulates the feed intake in ruminants. Although mOAPB are targeted at promoting intestinal health, it is not known whether additional benefits on the ruminal fermentation would be obtained from dietary supplementation of mOAPB. Albeit microencapsulation represents an effective strategy to decrease effects of microencapsulated products within the ruminal environment, Yoshimaru et al. (1999) reported that ~35% of microencapsulated products are usually released during ruminal fermentation, allowing it to influence the ruminal microbiome and fermentation.

We hypothesized that the utilization of mOAPB, with the goal of improving intestinal health, would have a ruminal side effect, modulating ruminal fermentation, improving it by acting as an antimicrobial agent in the rumen environment, selecting microorganisms that would improve ruminal fermentation. Thus, the objective of this study was to evaluate levels of mOAPB as dietary supplements in lactating dairy cow diets and their effects on nutrient degradability, nitrogen metabolism, and metabolites concentration in an in vitro ruminal fermentation simulator.

## Materials and Methods

All procedures using animals were approved by the University of Florida’s Institutional Animal Care and Use Committee.

### Experimental Design and Diets

Eight fermenters of a dual-flow continuous-culture system were used in a replicated 4 × 4 Latin square design with four treatments and four fermentation periods. Treatments (diets) were randomly assigned within Latin square for each period.

The basal diet was formulated to meet or exceed the requirements of a 680-kg Holstein dairy cow producing 45 kg of milk per day with 3.5% fat, 3.0% protein, and 4.8% lactose and an intake of 25 kg of dry matter (**DM**) per day (NASEM, 2021). The tested mOAPB (AviPlus P, Vetagro S.p.A., Italy) contained 55.6% hydrogenated and refined palm oil, 25.0% of citric acid, 16.7% of sorbic acid, 1.7% thymol, and 1.0% of vanillin. Treatments were arranged in levels of inclusion [0, 30, 60, and 90 g of product per 25 kg of total mixed ration (TMR)], where 30 g/25 kg of TMR is the recommended dose by the manufacturer and greater doses of the supplement were also tested. All experimental diets were formulated to have a similar nutritional composition (Table 1).

**Table 1.** Ingredient and chemical composition of experimental diets

Parameter^1^	Diet^2^
Basal diet, % DM	
Corn silage	47.2
Grass hay	14.5
Ground corn	15.5
Soybean meal	20.5
Calcium phosphate	0.19
Magnesium oxide	0.14
Calcium carbonate	1.43
Trace mineral premix^3^	0.50
Chemical composition, % DM	
CP	16.1
EE	2.43
NDF	30.9
ADF	17.8
Starch	32.0
Ca	0.67
P	0.37
Mg	0.25
K	1.21

^1^Expressed as percent of DM unless otherwise stated; CP, crude protein; EE, ether extract; NDF, neutral detergent fiber; ADS, acid detergent fiber; Ca, calcium; P, phosphorus; Mg, magnesium; K, potassium.

^2^Levels of inclusion of AviPlus P of 0, 30, 60, and 90 g/25 kg of total mixed ration.

^3^Composition of the trace mineral premix (% of the DM of the product): sodium (min) = 37.0%; sodium (max) = 38.7%; chloride (min) = 57.1%; chloride (max) = 59.8%; cooper (min) = 0.03%; zinc (min) = 0.35%; iron (min) = 0.20; manganese (min) = 0.01%; cobalt (min) = 0.01%.

The whole plant corn silage was collected and dried in a forced-air oven (Heratherm, Thermo Scientific) at 60 °C for 72 h. Subsequently, whole corn silage and the other feed ingredients, such as grass hay, corn meal, and soybean meal, were ground in a Wiley mill (model no. 2; Arthur H. Tomas Co.) to pass a 2-mm screen. Each ingredient was homogenized, and a subsample was collected in a labeled bag and sent to SDK Laboratories (Hutchinson, KS) for feed analysis.

### Animals and inoculum collection

Two ruminally cannulated lactating Holstein dairy cows were used as ruminal content donors. Cows averaged 229 DIM, 730 kg of body weight, 26.7 kg of milk yield, and were ruminally cannulated and kept at the Dairy Research Unit of the University of Florida; in a free-stall barn with the other cows of the herd receiving TMR containing 50% corn silage. On the first day of each experimental period, the ruminal content was collected from the cows 2 h after the morning feeding and filtered through four layers of cheesecloth and stored in a prewarmed thermos. The inoculum was transported within 30 min of the collection to the laboratory and incubated in the prewarmed dual-flow in vitro system.

### Dual-Flow Continuous-Culture System

The experiment was carried out in a dual-flow continuous-culture system similar to the one described by Hoover et al. (1976) and used in recent studies in our laboratory (Agustinho et al., 2022; Monteiro et al., 2022; Vinyard et al., 2023). Briefly, ruminal contents were collected from the ruminally cannulated lactating Holstein dairy cows, mixed in a ratio of 1:1 (v/v) from each cow, and poured into the eight prewarmed fermenter vessels. Each fermenter was fed twice a day at a constant rate of 106 g DM/d (53 g each meal). This system simulates the ruminal fermentation efficiently due to constant temperature at 39 ± 0.85 °C, agitation at 100 rpm, infusion of artificial saliva at 3.0 ± 0.11 mL/min, and constant infusion of N_2_ gas to maintain an anaerobic environment. The artificial saliva was formulated according to Weller and Pilgrim (1974) to allow the passage of liquid and solid flow. The outflow passage rate (K_p_) of the system across the experiment was 10.53 ± 0.45 %/h, where the liquid outflow contributed with 4.56 ± 0.25 %/h.

Each experimental period was 10 d in length, and every period the inoculum was replaced with a fresh one collected from the same donors. The first 7 d of each period was considered as an adaptation of the microbiome of the inoculum to the diet and system (Ziemer et al., 2000), and the last 3 d were used for sampling and data collection. On the first 5 d of each period of fermentation, ^15^N-nonenriched artificial saliva was used (Weller and Pilgrim, 1974). On day 5 of each experimental period, a 1-mL pulse dose of 0.0173 g/mL of 10.2% labeled (^15^NH_4_)_2_SO_4_ (Sigma-Aldrich Co.) was added directly into each fermenter and nonenriched saliva was replaced with an ^15^N-enriched saliva formulated to contain 0.077 g of 10.2% of labeled (^15^NH_4_)_2_SO_4_ per liter until day 10 of the fermentation. The ^15^N was used as a marker to estimate microbial protein synthesis (Calsamiglia et al., 1996).

### Sampling and Data Collection

On day 5 of each experimental period, before the change in the saliva, as described earlier, a sample of nonenriched saliva and background digesta from each fermenter were collected to determine the DM, ash, and ^15^N abundance. Samples were stored at −20 °C for further processing and analysis.

For the evaluation of the kinetics of the metabolic activity of each fermenter, samples, and measurements from the ruminal content within each fermenter were collected. The pH of the ruminal content of each fermenter was measured at 0, 1, 2, 4, 6, and 8 h after the morning feeding (0800 hours) on days 8, 9, and 10 of the experimental periods using a portable pH meter (Thermo Scientific Orion Star A121, Thermo Fisher Scientific Inc., MA). The pH meter was calibrated on each experimental period using standard pH buffer 4, 7, and 10 (SB105, Thermo Fisher Scientific Inc.).

On days 9 and 10 of each experimental period at 0, 1, 2, 4, 6, and 8 h after the morning feeding (0800 hours), ~10 mL of ruminal content was manually collected from each vessel. The samples were filtered through four layers of cheesecloth and stored into 15 mL tubes. These samples were acidified with 100 µL of 50% H_2_SO_4_ (v/v) within 10 min of the collection and stored at −20 °C for further processing and short-chain fatty acids (SCFA) and NH_3_–N analysis. At the same time of the previously described collection, an extra 1.5 mL ruminal sample was collected from each fermenter and stored into a 2-mL microcentrifuge tube (Fisherbrand, CA) at −20 °C for further processing and lactate analysis. Kinetics of pH and fermentation metabolites concentration are reported in the table as the LSMeans result across days and timepoints.

The digesta effluent containers, which consisted of two separate containers, one to store the liquid effluent outflow and another to store the solid effluent outflow, were kept in a cold-water bath (4 °C) on days 9 and 10 to inhibit further fermentation of the effluent outflow. Effluent of the liquid and solid outflow was pooled daily within fermenter. Duplicate subsamples of 180 g of the pooled effluent from each fermenter were collected daily and stored at −20 °C for further processing and nutrient analysis. At the same time, another sample of the pooled effluents was collected and filtered through four layers of cheesecloth. A 40-mL sample was then collected and acidified with 400 µL of 50% H_2_SO_4_ (v/v) for SCFA analysis and another 1.5 mL nonacidified sample was collected for lactate analysis.

At the end of the experimental period, the entire content from each fermenter was collected for the isolation of the bacteria. The following procedure used for bacterial isolation was modified from that described by Krizsan et al. (2010). The total ruminal content from each vessel was collected and blended for 30 s with 200 mL of 0.9% saline solution using a household blender, squeezed through four layers of cheesecloth, and rinsed with another 200 mL of 0.9% saline solution. The filtrate was then centrifuged (Allegra X-15R Centrifuge, Beckman Coulter Life Sciences, CA) at 1,000 × *g* for 10 min at 4 °C to remove the residual feed particles. The supernatant was then collected and centrifuged in an ultraspeed centrifuge (Sorvall RC-5B Refrigerated Superspeed Centrifuge, DuPont Instruments, DE) at 11,250 × *g* for 20 min at 4 °C for isolation of the bacterial pellet. The supernatant was carefully discarded and the bacterial pellet was resuspended using 200 mL of McDougall’s solution (McDougall, 1948). The resuspended solution was then centrifuged (Sorvall LYNX 4000 Centrifuge, Thermo Scientific, GA) at 16,250 × *g* for 20 min at 4 °C. The bacterial pellet was harvested from the third centrifugation and stored at −20 °C for further processing and analysis.

### Laboratory Analysis

Acidified ruminal samples that were collected for SCFA and NH_3_–N concentration analysis were centrifuged (Sorvall LYNX 4000 Centrifuge, Thermo Scientific) at 10,000 × *g* for 15 min at 4 °C. NH_3_–N concentration was determined in duplicate according to Broderick and Kang (1980) and adapted to a plate reader by using 2 µL of the sample, 100 µL of phenol solution, and 80 µL of hypochloride solution in each well of the microplate. Absorbance was measured in a spectrophotometer (SpectraMax Plus 384 Microplate Reader, Molecular Devices, San Jose, CA) at 620 nm. The inter- and intra-assay coefficients of variability of the analysis were 2.85 and 5.37%, respectively.

Samples to determine SCFA were further processed, according to Ruiz-Moreno et al. (2015), where solution of 2 g/L (w/v) of crotonic acid and 25% (w/v) of metaphosphoric acid was added in a 1:5 ratio (crotonic and metaphosphoric acid to sample) to the supernatant and frozen overnight. The sample was then centrifuged at 10,000 × *g* for 15 min at 4 °C. The supernatant was recovered, and ethyl acetate was added in a 2:1 ratio to the supernatant, vortexed, and allowed to separate into layers, according to Ruiz-Moreno et al. (2015) with the following modifications. The top layer was transferred to a chromatography vial. The concentration of acetate, propionate, butyrate, isobutyrate, isovalerate, valerate, and caproate was determined by gas chromatography (Agilent 7820A GC, Agilent Technologies, Shanghai, China) using a flame ionization detector and a capillary column (CP-WAX 58 FFAP 25 m 0.53 mm, Varian CP7767, Varian Analytical Instruments, CA) at 110 °C with injector temperature at 200 °C and detector at 220 °C.

Lactate concentration was determined using a commercial kit (d-Lactic acid/l-Lactic acid kit, R-Biopharm AG, Darmstadt, Germany). Samples were processed and analyzed according to the instructions provided by the manufacturer. Absorbance was measured in a spectrophotometer (SpectraMax Plus 384 Microplate Reader, Molecular Devices). The inter- and intra-assay coefficients of variability of the analysis were 8.87% and 5.17%, respectively.

Background saliva, background digesta, bacterial pellet, and digesta samples were freeze dried (FreeZone 6, Labconco, MO). All of these samples and the feed ingredients were then analyzed for DM content, using an air-forced oven at 105 °C for 12 h, according to the method no. 930.15 (AOAC, 1990). Ash was also determined, using a muffle furnace (Isotemp Muffle Furnace, Fisher Scientific, GA) at 600 °C for 6 h, according to the method no. 942.05 (AOAC, 1990). A dry subsample of about 0.5 mL (volume) of each of these samples was collected and placed into a 2-mL impact-resistant microcentrifuge tubes (Fisherbrand, CA) and 0.5 mL (volume) of 2 mm zirconia beads (BioSpec Product, Bartlesville, OK) was added, the tube was properly labeled and closed. The sample was then pulverized in a homogenizer (Precellys 24, Bertin, Montigny-le-Bretonneux, France) at 5,500 × *g* for 10 s for determination of total nitrogen and ^14^N and ^15^N partitioning.

The samples were loaded into tin capsules (Elemental microanalysis, Okehampton, United Kingdom) and weighed using a microscale (Excellence Plus XP Micro Balance Mettler-Toledo GmbH, Laboratory & Weighing Technologies, Mississauga, Canada). To avoid NH_3_–sN contamination during the analysis, 35 µL of K_2_CO_3_ solution (10 g/L) was added to the samples and allowed to dry overnight in a forced-air oven at 40 °C. The percent ^15^N in dried samples was determined using a mass spectrometer (IsoPrime 100, IsoPrime, Langenselbold, Germany), and the results were expressed as the fractional abundance of isotopic fractions (^15^N/^14^N).

For all samples, except for the bacterial pellet, NDF was analyzed following the procedure described by Mertens (2002) with the addition of thermostable α-amylase, sodium sulfite, and corrected for ash in an Ankom200 Fiber Analyzer (Ankom Technology, Macedon, NY).

### Calculations for Disappearance of Nutrients and N Metabolism

Nutrient disappearance in the digesta effluent was estimated according to Soder et al. (2013), as follows:


$$\text{ND}=\;100\;\times \;\frac{\text{NI}-\left(\text{NE}+\text{NS}+\text{NB}\right)}{\text{NI}}$$

where ND is the percentage of nutrient disappearance in a DM basis, NI is the nutrient intake (g/d), NE is the nutrient outflow in the effluent (g/d), NS is the nutrient in the saliva (g/d), and NB is the nutrient in the bacterial pellet (g/d).

Total N present in the digesta effluent corresponds to the N remaining from the microbial fermentation, and it was subdivided into NH_3_–N flow (**ANF**), dietary N flow, and bacterial N flow (**BNF**). The nonammonia nitrogen (**NAN**) is a combination of the dietary N flow and bacterial N flow. These parameters were calculated according to Calsamiglia et al. (1996) and Bach and Stern (1999):


$${\text{NH}}_{3}\text{N flow}={\text{NH}}_{3}\;\times \;\frac{\text{TE}}{100}$$

$$\text{NAN}=\text{TN}-{\text{NH}}_{3}\text{N flow}$$

$$\text{Bacterial N flow}=\;\frac{\text{NAN}\;\times {\text{NAN}}^{15}\text{N}}{{\text{B}}^{15}\text{N}}$$

where NH_3_N flow is the ammonia nitrogen flow (g/d); NH_3_ is the NH_3_–N concentration in the effluent (mg/dL); TE is the volume of total effluent flow (mL); NAN is the nonammonia nitrogen flow (g/d); TN is the total nitrogen in the effluent (g); bacterial N flow is the bacterial nitrogen flow (g/d); NAN^15^N is the percentage of atom excess of ^15^N in NAN, which is the % atom ^15^N in NAN effluent sample minus the % atom ^15^N in the background sample (%); B^15^N is the percentage of atom excess of ^15^N in the bacterial pellet.

In addition, the flow of dietary N and microbial efficiency indicators were determined according to Bach and Stern (1999):


Dietary N flow= NAN−Bacterial N flow

$$\text{BE}=\frac{\text{Bacterial N flow}}{\text{tOMD}}$$

$$\text{ENU=}\;\frac{\text{BNF}}{N}\;\times 100$$

where dietary N flow is the dietary nitrogen flow (g/d); NAN is the nonammonia nitrogen flow (g/d); bacterial N flow is the bacterial nitrogen in effluent (g/d); BE is the bacterial efficiency (g of bacterial N/kg of tOMD); BNF is the bacterial nitrogen flow (g/d); tOMD is the truly organic matter degraded (kg); ENU is the efficiency of nitrogen utilization (%); BNF is the bacterial nitrogen flow (g/d); N is the total nitrogen available (g/d).

### Statistical Analysis

The experiment was conducted in a duplicated 4 × 4 Latin square, where the fermenter within Latin square was the experimental unit. Normality of residuals and homogeneity of variance were examined for each continuous dependent variable using the Shapiro–Wilk test from the UNIVARIATE procedure of SAS 9.4 (SAS Institute Inc., Cary, NC). All the variables, except nitrogen metabolism variables, were analyzed as repeated measurements using the Compound Symmetry covariance structure, the covariance structure was selected based on the smallest corrected Akaike’s information criterion after model fitting.

Statistical analysis for the nutrient disappearance, N metabolism, and SCFA concentration in the daily outflow was performed using the MIXED procedure of SAS 9.4, using the model:


$$Y_{ijklm}=\text{ ​μ}+T_{i}+D_{j}+P_{k}+S_{l}+S{\left(F\right)}_{lm}+e_{ijklm}$$

where *Y*_*ijklm*_ is the observation *ijklm*; µ is the overall mean; *T*_*i*_ is the fixed effect of treatment (*i* = 1 to 4); *D*_*j*_ is the random effect of day of collection (j = 1 to 2); *P*_*k*_ is the random effect of period (*k* = 1 to 4); *S*_*l*_ is the random effect of square (l = 1 to 2); *S(F)*_*km*_ is the random effect of fermenter (*m* = 1 to 8) nested within Latin square (l = 1 to 2); and *e*_*ijklm*_ is the random residual. The evaluation of the kinetics of SCFA, NH_3_–N, and lactate was carried out using the model:


$$Y_{ijklmn}=\text{​ μ }+T_{i}+U_{j}+T_{i}\times U_{j}+D_{k}+P_{l}+S_{m}+S{\left(F\right)}_{nm}+e_{ijklmn}$$

where *Y*_*ijklmn*_ is the observation *ijklmn*; µ is the overall mean; *T*_*i*_ is the fixed effect of treatment (*i* = 1 to 4); *U*_*j*_ is the fixed effect of time (*j* = 1 to 6); *T*_*i*_ × *U*_*j*_ is the fixed effect of the interaction of the treatment (*i* = 1 to 4) and time (*j* = 1 to 6); *D*_*k*_ is the random effect of day of collection (*j* = 1 to 2); *P*_*l*_ is the random effect of period (*l* = 1 to 4); *S*_*m*_ is the random effect of square (*l* = 1 to 2); *S(F)*_*nm*_ is the random effect of fermenter (*n* = 1 to 8) nested within Latin square (*m* = 1 to 2); and *e*_*ijklmn*_ is the random residual. The effect of levels of inclusion of the mOAPB was depicted by orthogonal contrasts (linear and quadratic). Significance was declared at *P* ≤ 0.05, and tendency was declared at 0.05 < *P* ≤ 0.10.

## Results

The dietary levels of supplementation of mOAPB in diets of lactating dairy cows were studied in vitro and their effects on ruminal fermentation were evaluated. For the kinetics parameters, where statistical model included time and time by treatment as fixed effect, all the studied variables were significant for time (*P *< 0.01, data not shown) and no interaction between treatment and time was observed (*P* > 0.05, data not shown), thus effects of the interaction between treatment and time will not be further discussed.

**Figure 1.**
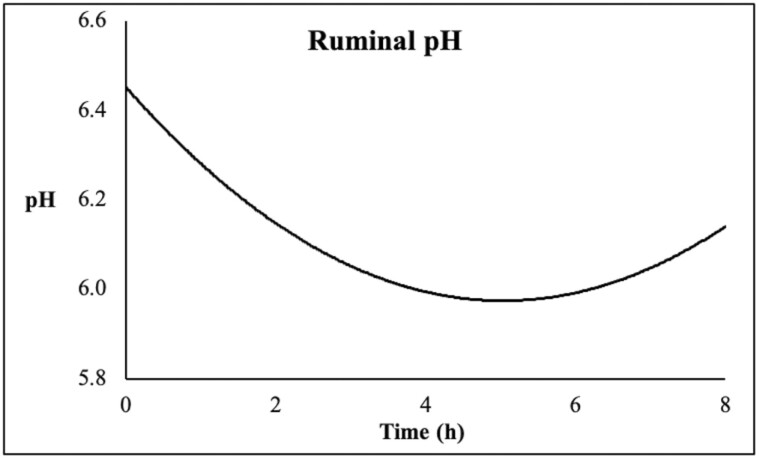
Daily pH fluctuation with fermenter across all treatments and periods from feeding time starting at 0 up to 8 h post-feeding.

In Table 2, we can observe the fermentation metabolites outflow concentration. There was a positive linear effect of the level of inclusion of mOAPB on lactate (*P* = 0.01) and l-lactate (*P* < 0.01) concentration of the daily outflow. The d-lactate was the predominant form of lactate in the sample, comprising 55.5% of the total lactate and there was no effect of the treatment. There was no effect of the level of inclusion of mOAPB on the molar concentration of the SCFAs and molar proportion of SCFAs. In Table 3, we can observe the kinetics of pH and fermentation metabolites. There was no effect of the treatment or treatment and time interaction (*P* > 0.10). In Fig. 1, we can observe the daily pH variation across all treatments and periods. There was a variation between 6.45 at the morning feeding time and 5.98 around 5 h after morning feeding. In Table 4, we can observe the apparent and true degradability of nutrients. There were no effects of the degradability of the nutrients evaluated. In Table 5, we can observe the N metabolism variables. There was no effect of treatment on the N metabolism (*P* > 0.10).

**Table 2.** Effect of microencapsulated organic acids and pure botanicals on fermentation metabolites of daily effluents (24 h) in a dual-flow continuous-culture system

Parameter	Level of inclusion^1^				SEM	*P*-value^2^	
	0	30	60	90		Lin	Quad
Lactate, mM/dL	0.11	0.12	0.12	0.14	0.02	0.01	0.61
d-lactate, mM/dL	0.06	0.07	0.06	0.08	<0.01	0.10	0.34
d-lactate, %	54.0	56.2	56.0	55.9	1.74	0.55	0.56
l-lactate, mM/dL	0.05	0.05	0.06	0.06	<0.01	<0.001	0.99
l-lactate, %	46.0	43.8	44.0	44.1	1.74	0.55	0.56
SCFA, mM/L							
Acetate	46.0	46.0	46.2	47.6	1.36	0.22	0.46
Propionate	19.1	19.8	19.4	19.4	0.41	0.84	0.49
Butyrate	17.5	17.0	17.9	16.9	1.24	0.80	0.62
Isobutyrate	0.77	0.76	0.82	0.80	0.05	0.14	0.65
Isovaleric	2.48	2.38	2.72	2.58	0.26	0.42	0.92
Valeric	2.32	2.21	2.31	2.31	0.13	0.84	0.45
Caproic	3.08	3.21	2.86	3.16	0.25	0.88	0.61
Total	92.3	91.4	92.2	92.7	2.34	0.69	0.56
A/P	2.41	2.35	2.41	2.47	0.08	0.50	0.39
A + B/P	3.33	3.22	3.34	3.35	0.13	0.68	0.55
Molar proportion							
Acetate	50.4	50.4	50.1	51.3	0.50	0.36	0.27
Propionate	21.0	21.6	20.8	20.6	0.53	0.35	0.33
Butyrate	19.1	18.6	19.4	18.2	0.89	0.46	0.50
Isobutyrate	0.84	0.84	0.89	0.86	0.04	0.22	0.53
Isovaleric	2.72	2.60	2.95	2.78	0.28	0.48	0.88
Valeric	2.55	2.42	2.50	2.49	0.14	0.67	0.25
Caproic	3.40	3.53	3.11	3.41	0.30	0.60	0.61

^1^Levels of inclusion ranging from 0 to 90 g/25 kg of total mixed ration.

^2^Contrast: linear and quadratic, there was no cubic effect, thus *P*-values are not presented.

**Table 3.** Effect of microencapsulated organic acids and pure botanicals on kinetics of pH and fermentation metabolites in a dual-flow continuous-culture system collected at 0, 1, 2, 4, 6, and 8 h postmorning feeding

Parameter	Level of inclusion^1^				SEM	*P*-value^2^	
	0	30	60	90		Lin	Quad
pH	6.11	6.16	6.14	6.12	0.04	0.92	0.16
NH_3_, mM/dL	12.3	11.6	13.0	12.2	0.84	0.56	0.76
Lactate, mM/dL	0.06	0.07	0.08	0.09	0.01	0.18	0.99
d-lactate, mM/dL	0.04	0.04	0.05	0.05	0.01	0.18	0.91
d-lactate, %	62.8	59.3	61.2	60.6	1.26	0.24	0.12
l-lactate, mM/dL	0.02	0.03	0.03	0.03	0.01	0.21	0.86
l-lactate, %	37.2	40.7	38.8	39.4	1.26	0.24	0.12
SCFA, mM/L							
Acetate	45.6	44.6	45.3	46.2	0.82	0.37	0.12
Propionate	18.7	19.2	18.6	18.8	0.61	0.95	0.67
Butyrate	16.5	15.8	16.7	15.7	1.31	0.57	0.79
Isobutyrate	0.82	0.77	0.86	0.81	0.04	0.50	0.68
Isovaleric	2.47	2.26	2.66	2.46	0.21	0.60	1.00
Valeric	2.34	2.13	2.27	2.21	0.08	0.41	0.29
Caproic	2.96	2.88	2.65	2.88	0.23	0.51	0.35
Total	89.3	87.7	89.2	88.9	2.05	0.90	0.50
A:P^3^	2.45	2.33	2.43	2.47	0.08	0.59	0.27
(A + B):P^2^	3.34	3.15	3.33	3.32	0.14	0.77	0.37
Molar proportion, %							
Acetate	56.5	55.4	56.1	57.4	1.00	0.33	0.12
Propionate	23.2	23.9	23.1	23.4	0.76	0.95	0.67
Butyrate	20.5	19.6	20.8	19.5	1.63	0.57	0.79
Isobutyrate	1.01	0.96	1.07	1.00	0.05	0.50	0.68
Isovaleric	3.06	2.81	3.31	3.05	0.27	0.61	0.99
Valeric	2.90	2.65	2.82	2.74	0.10	0.41	0.29
Caproic	3.68	3.58	3.28	3.56	0.28	0.48	0.36

^1^Levels of inclusion ranging from 0 to 90 g/25 kg of total mixed ration.

^2^Contrast: linear and quadratic, there was no cubic effect, thus *P*-values are not presented. Fixed effect of time for all variables was significant (*P* < 0.001), the interaction between treatment and time was not significant for all analyzed variables (*P* > 0.05), thus *P*-values will not be shown.

^3^A:P is the molar ratio of acetate and propionate; (A + B):P is the ratio of acetate and butyrate divided by propionate.

**Table 4.** Effect of microencapsulated organic acids and pure botanicals on ruminal degradability of DM, OM, CP, NDF, and ADF in a dual-flow continuous-culture system

Parameter^1^, %	Level of inclusion^2^				SEM	*P*-value^3^	
	0	30	60	90		Lin	Quad
Apparent degradability							
DM	55.8	54.1	55.8	54.1	2.47	0.58	0.98
OM	49.8	48.3	49.5	48.3	2.11	0.58	0.91
CP	58.0	58.8	59.8	59.8	3.87	0.73	0.92
True degradability							
DM	68.6	65.2	67.9	67.5	2.07	0.93	0.17
OM	60.9	57.9	60.0	59.6	1.80	0.75	0.23
CP	63.6	59.9	60.5	61.9	2.34	0.69	0.32
NDF	54.3	56.1	52.3	53.4	2.90	0.48	0.86
ADF	44.6	43.7	44.3	43.3	4.85	0.80	0.99

^1^Apparent degradability is the percentage of the nutrient degraded and true degradability is the percentage of the nutrient degraded discounting for the nutrient present in the bacterial pellet. DM, dry matter; OM, organic matter; CP, crude protein; NDF, neutral detergent fiber; ADF, acid detergent fiber.

^2^Levels of inclusion ranging from 0 to 90 g/25 kg of total mixed ration.

^3^Contrast: linear and quadratic, there was no cubic effect, thus *P*-values are not presented.

**Table 5.** Effect of microencapsulated organic acids and pure botanicals on N metabolism in a dual-flow continuous-culture system

Parameter^1^, %	Level of inclusion^2^				SEM	*P*-value^3^	
	0	30	60	90		Lin	Quad
NH_3_–N, mg/dL	4.49	4.11	4.47	4.41	0.48	0.91	0.49
Total N^4^	2.35	2.38	2.41	2.41	0.11	0.68	0.84
NH_3_–N^5^	0.19	0.18	0.20	0.19	0.02	0.81	0.93
NAN^6^	2.15	2.20	2.21	2.21	0.11	0.69	0.85
Bacteria N^7^	1.16	1.10	1.12	1.16	0.07	0.88	0.42
Dietary N^8^	1.00	1.10	1.09	1.05	0.06	0.63	0.32
ENU^9^	41.9	45.7	44.8	43.9	1.45	0.48	0.15
Bacterial efficiency^10^	19.3	19.6	18.8	19.6	1.43	0.95	0.83
RDP-N supply^11^	2.50	2.40	2.43	2.47	0.06	0.78	0.37
RDP-N supply^12^	71.5	68.7	69.1	70.1	1.83	0.66	0.34
RUP-N supply^11^	0.99	1.10	1.09	1.05	0.06	0.63	0.32
RUP-N supply^12^	28.5	31.3	30.9	29.9	1.83	0.66	0.34

^1^Apparent degradability is the percentage of the nutrient degraded and true degradability is the percentage of the nutrient degraded discounting for the nutrient present in the bacterial pellet.

^2^Levels of inclusion ranging from 0 to 90 g/25 kg of total mixed ration.

^3^Contrast: linear and quadratic, there was no cubic effect, thus *P*-values are not presented.

^4^Total N =  total N flow (g/d) = NH_3_-N + NAN (Bach and Stern, 1999).

^5^NH_3_–N = ammonia N flow (g/d) = mg/dL of effluent NH_3_–N × (g of total effluent flow/100).

^6^NAN = nonammonia N flow (g/d) = total N − NH_3_-N.

^7^Bacterial-N flow (g/d) = (NAN flow × % atom excess of 15N in NAN effluent)/(% atom excess of ^15^N in bacteria pellet), according to Calsamiglia et al. (1996).

^8^Dietary N flow (g/d) = g of effluent NAN − g of effluent bacterial N.

^9^ENU = efficiency of N use = (g of bacterial N/g of available N) × 100 (Bach and Stern, 1999).

^10^Bacterial efficiency = g of bacterial N flow/kg of OM truly digested (Calsamiglia et al., 1996).

^11^N supply = g/d.

^12^N supply %.

## Discussion

Blends of OA and PB have been widely used as a feed additives in the monogastric livestock industry, as an alternative to growth-promoting antibiotics (EFSA, 2012; Tugnoli et al., 2020). Their utilization in ruminants has been limited because of potential ruminal effects or ruminal degradation, which would limit their post-ruminal availability. Microencapsulation of OA and PB would potentially minimize or eliminate ruminal effects and ruminal degradation, potentially allowing these compounds to escape ruminal degradation and be available for digestion and absorption in the small intestine. Organic acids are traditionally applied as a food preservative and have a demonstrated efficacy as antimicrobials, due to their broad spectrum of application, such as antibacterial (Grilli et al., 2013), antiviral (Cochrane et al., 2016), and antifungal (Leyva Salas et al., 2017) properties. However, not all OAs have antimicrobial activity, this property is highly dependent on the carbon chain length and degree of unsaturation, which drive the pK_a_, which represents the pH where 50% of the acid is found in a dissociated form, of the acid and its mechanism of action (Huyghebaert et al., 2011).

For instance, based on the p*K*_a_ of the OA and pH of the environment, the OA can be in its undissociated form and diffuse across the bacterial cell membrane and dissociate in the cytoplasm, which releases H^+^ ions and decreases intracellular pH (Russell and Diez-Gonzalez, 1997). As an attempt to overcome the reduction of intracellular pH, microorganisms that are not able to maintain their metabolic activity within a range of intracellular pH activates ATP mediated proton pumps at the cell membrane, concomitantly, the dissociated OA disrupt the normal DNA replication, leading to bacteriostatic or bactericidal effects (Russell and Wilson, 1996; Lambert and Stratford, 1999).

Similarly, PB have a wide variety of application, with some compounds having important biological activities including anti-inflammatory, antioxidant, antimicrobial, and immunomodulatory (Rossi et al., 2020). More specifically, thymol and vanillin have been studied and both plant botanicals have a strong antimicrobial activity (Burt et al., 2007; Ponnusamy et al., 2009); however, more recently, both PB have been tested and reported to have strong antioxidant and anti-inflammatory properties (Rossi et al., 2020). For instance, protected blend of thymol and its isomer carvacrol, when tested on *Clostridium perfrigens*-challenged broilers as a dietary additive at levels ranging from 0 to 240 mg/kg, linearly alleviated gut lesions and improved the ratio of villus height to crypt depth. In addition, there was a linear increase in feed conversion and decrease in inflammatory response by reduction of the mRNA expression of toll-like receptor 2 and tumor necrotic factor-α in the ileum of the broilers (Du et al., 2016).

Likewise, studies have shown that vanillin can also reduce gut inflammation, by downregulating the mucosa expression of pro-inflammatory cytokines such as interleukin-1β, interleukin-6, interferon-γ, and tumor necrosis factor-α (Jung et al., 2008; Ciciliato et al., 2022). In addition, vanillin has antioxidant potential, which leads to inhibition of protein oxidation and lipid peroxidation, increasing antioxidant enzymes and scavenges free radical, which consequently protects the DNA and mitochondrial membrane against oxidative stress (Kumar et al., 2004; Tai et al., 2011). Since most of the OA and PB have beneficial activity in the intestine, protection or encapsulation techniques should be used to protect these active compounds from microbial fermentation and enzymatic activity.

Piva et al. (1997) studied the microencapsulation of tryptophan and sulfamethazine and reported that a protective matrix delayed absorption without affecting total bioavailability of the protected compounds, suggesting a prolonged effect on the release of the compound. In another study, evaluating the microencapsulation of organic acids for pigs, Piva et al. (2007) reported that microencapsulation of OA allowed a reduction in the additive dose by 10-fold to obtain a comparable production response, such as body weight and mortality index. In addition, the utilization of microencapsulation strategies enables the utilization of OA and PB that target the intestine in ruminants (Kim et al., 2020; Fontoura et al., 2022, 2023).

Due to these benefits of OA, PB, and the blend of those active compounds; these have been successfully used in livestock nutrition as antimicrobial and immunomodulator of the gut, especially in a wide variety of monogastric species, such as sea bass (Busti et al., 2020), rainbow trout (Pelusio et al., 2020), broilers (Bialkowski et al., 2023; Stingelin et al., 2023), and pigs (Grilli et al., 2015; Hutchens et al., 2021). More recently, an interest in evaluating the effect of mOAPB in ruminants has increased. In the study by Fontoura et al. (2023), the mOAPB (same as used in this trial) were used as feed additive in calves challenged by heat stress. The author reported that low level of mOAPB (75 mg/kg of body weight) was able to partially restore dry matter intake (**DMI**) for calves during heat stress on the first week of exposure (3.39 vs. 2.81 kg of DMI), which indicates a potential strategy to improve intake in dairy calves exposed to acute bouts of heat stress.

In a follow-up experiment, Fontoura et al. (2022) evaluated the effects of the same mOAPB in lactating dairy cows during a heat stress challenge. Heat stress can negatively affect the production of lactating dairy cows, by—among other issues—reducing dry matter intake and consequently reducing milk yield (Kim et al., 2022). In addition, heat stress can cause infiltration of the mucosa and submucosa of the jejunum by endotoxins (such as lipopolysaccharides), which leads to systemic inflammation (Koch et al., 2019). Fontoura et al. (2022) reported that mOAPB during heat stress enabled a 10% DMI increase when compared to the control heat-stressed animals, which resulted in a significant increase in milk yield (22.5 vs. 25.2 kg/d) and energy-corrected milk (29.0 vs. 31.5 kg/d). In the same study, authors reported lower plasma concentrations of lipopolysaccharide-binding protein (endotoxin biomarker) in cows supplemented with mOAPB compared to control. The underlying mechanism by which mOAPB increase intake in ruminants is uncertain; however, the authors suggested that intestinal insulin growth factor system activation by sorbic acid may play a role.

Because of the consistent beneficial effects experimentally demonstrated in the lower gastrointestinal tract (small intestine) and to the microbial modulator action of those active compounds (Feye et al., 2020; Pelusio et al., 2020), it was hypothesized that a small fraction of OA and PB could be released in the rumen and modulate the microbial fermentation. Due to the fact that microencapsulation usually releases ~35% of its product of interest during the ruminal fermentation (Yoshimaru et al., 1999). In our study, we did not observe major differences between the control and mOAPB supplemented fermenters even with a supplementation three times greater than the basal recommended supplementation. This increases the likelihood of the active compounds to be effectively protected against ruminal fermentation and be available at the intestinal level. The lack of effects of the biomolecules in the fermentation jar could be attributed to the pH within the fermenter (daily variation between 6.45 and 5.98). The pH of the aqueous solution plays a major role in the interfacial tension between the solution and the lipid, where greater pH can potentialize the emulsification and solubility of fatty acids (Hsieh et al., 2021). Thus, further studies are required to test the effectiveness of fat-microencapsulation technologies in a range of pHs.

Amin et al. (2021) evaluated the disappearance of free and microencapsulated essential oils in in vitro ruminal fermentation. It was reported that essential oils microencapsulated using a matrix of vegetable hydrogenated fatty acids, calcium carbonate, and starch (corn or wheat) enhance the stability of essential oils in the ruminal content, which enabled a recovery of about 90% of essential oils after 48 h of incubation. Such a high efficiency to escape the rumen enabled the release of most of these compounds in the intestine (Kim et al., 2020).

## Conclusions

In previous studies, unprotected organic acids and pure botanicals have been shown to improve health status in monogastric animals. Microencapsulated forms of these compounds have also shown positive effects in ruminants. Our focus was to evaluate the effects of a possible partial release of these compounds in the rumen and to achieve that, greater doses were tested. Overall, our results showed that there were no major effects of the microencapsulated active compounds on the kinetics of fermentation (pH) and metabolite concentration, such as volatile fatty acids and ammonia. Our results demonstrate that within physiological ruminal pH, the microencapsulation was effective to protect the mOAPB against ruminal fermentation, decreasing the chances for additional effects of the mOAPB in the ruminal environment. This suggests that these compounds escape ruminal fermentation and may become available for digestion and absorption in the small intestine.
